# Strategies for mitigating adverse events related to selective RET inhibitors in patients with RET-altered cancers

**DOI:** 10.1016/j.xcrm.2023.101332

**Published:** 2023-12-19

**Authors:** Mirella Nardo, Mohamed A. Gouda, Blessie E. Nelson, Carmelia M.N. Barreto, J. Hoyt Slade, Anna Poullard, Mark Zafereo, Mimi I. Hu, Maria E. Cabanillas, Vivek Subbiah

**Affiliations:** 1 Department of Investigational Cancer Therapeutics, The University of Texas MD Anderson Cancer Center, Houston, TX, USA; 2 Department of Head and Neck Surgery, The University of Texas MD Anderson Cancer Center, Houston, TX, USA; 3 Department of Endocrine Neoplasia and Hormonal Disorders, The University of Texas MD Anderson Cancer Center, Houston, TX, USA; 4 Pharmacy Clinical Programs, The University of Texas MD Anderson Cancer Center, Houston, TX, USA; 5 Sarah Cannon Research Institute, Nashville, TN, USA

**Keywords:** RET inhibitors, toxicity

## Abstract

The US Food and Drug Administration (FDA) approval of the selective RET inhibitors selpercatinib and pralsetinib has led to a paradigm change in the treatment of *RET*-altered lung and thyroid cancers through a higher response rate and a more tolerable safety and toxicity profile than multi-kinase inhibitors. Recently, selpercatinib has received a tissue-agnostic FDA approval for all *RET*-fusion-positive cancers, and pralsetinib has shown pan-cancer activity as well. Given the anticipated increase in the use of both drugs across multiple tumor types, it is crucial to recognize the possible side effects and approaches for their optimal management in order to maximize the clinical benefit for treated patients. In this review, we underscore potential toxicities associated with selective RET inhibitors and discuss strategies to mitigate them.

## Graphical abstract

**Figure:**
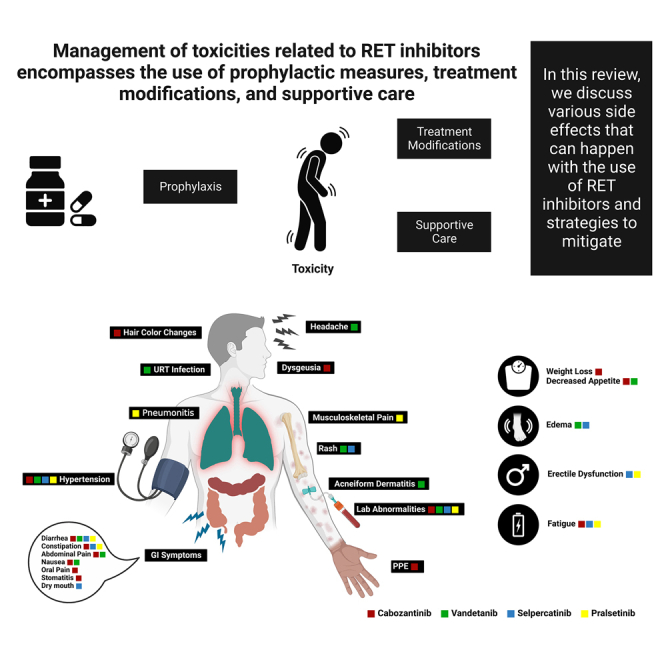

## Teaser

Advances in the RET inhibition field, including the approval of a selective RET inhibitor, necessitates proper understanding of associated side effects and strategies to mitigate. In this review, Nardo M. et al. elaborate on different toxicities associated with RET inhibitors and discuss approaches for clinical management.

## Introduction

**Figure 1:**
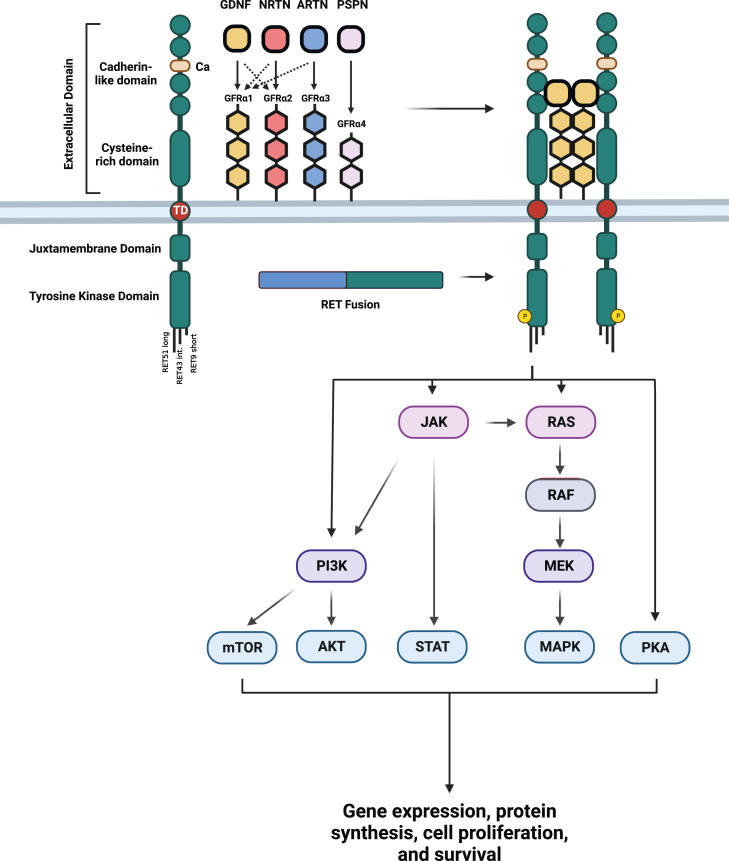
RET pathway This can be activated through ligand binding to extracellular co-receptor that leads to RET dimerization and phosphorylation of the tyrosine kinase domain which can activate multiple downstream pathways. RET fusion creates a chimeric oncoprotein with constitutive activation of its kinase domain.

Alterations in the *RET* gene have been described in different cancer types and can lead to uncontrolled activation of multiple proliferative signaling pathways (Figure 1). Therefore, targeting *RET* alterations has been an area of interest in the era of precision oncology. Multi-kinase inhibitors, including cabozantinib and vandetanib, with ancillary activity on RET have been developed.^1^ However, response rates and durability remained relatively low, and toxicity was substantially high because of off-target side effects from VEGFR2 and SRC inhibition.^1^^,^^2^ Besides, multi-kinase inhibitors were primarily designed to target other kinases and their partial inhibition of RET was apparently less potent. Additionally, those were inactive against *RET* gatekeeper mutations, which are not infrequent in patients with *RET*-altered cancers.^3^^,^^4^ The highly selective and potent RET-inhibiting agents, selpercatinib and pralsetinib were, therefore, developed to overcome some of the limitations of the multi-kinase inhibitors.^5^^,^^6^ Those drugs led to a paradigm shift in the treatment of *RET*-altered cancers, with higher response rates and more tolerable toxicity profile.^7^ Selpercatinib and pralsetinib have received regulatory approvals in RET-altered non-small cell lung cancer (NSCLC) and thyroid cancer. More recently, selpercatinib has received accelerated US Food and Drug Administration (FDA) approval for *RET*-fusion-positive cancers in a tissue-agnostic indication.^8^ Pralsetinib has also shown activity in multiple *RET*-fusion-positive solid tumors,^9^ and the widespread use of selpercatinib and pralsetinib necessitates proper understanding of potential toxicities and strategies to mitigate them.

Herein, we review the side effects profile of selective RET inhibitors and approaches to mitigate those toxicities in real-world practice. Recommendations are provided on the basis of a literature review and the expertise of our group in managing more than 100 patients with *RET*-altered cancers who were treated with selective RET inhibitors.

## Selective RET inhibitors

Selpercatinib received an accelerated approval from the FDA in May 2020 for treatment of patients with metastatic *RET*-fusion positive NSCLC, advanced/metastatic *RET*-mutant medullary thyroid cancer, and advanced/metastatic *RET*-fusion-positive thyroid cancer.^10^^,^^11^^,^^12^ In 2022, selpercatinib received full FDA approval for NSCLC and accelerated approval in a tissue-agnostic manner for all cancers harboring *RET* fusions. Similarly, pralsetinib was approved in September 2020 for NSCLC and in December 2020 for thyroid cancer.^13^

The pre-clinical to clinical development of these agents in record timeline and regulatory approvals represented a major step for the treatment of these patient populations, not only because of the better overall response rate (ORR) but also because of their better toxicity profile compared with multi-kinase inhibitors previously used in the treatment of *RET* dependent cancers. Moreover, the potency of the selective RET inhibitors in control of the central nervous system metastases was important, especially in patients with NSCLC.^14^ Table S1 shows the ORRs of selpercatinib and pralsetinib among the regulatory approvals’ published clinical trials.

## Pathophysiology of adverse events

Adverse events resulting from the use of RET inhibitors happen because of the inhibition of either endogenous RET or non-RET receptors. For example, there are data suggesting that hypersensitivity and QT interval prolongation with selpercatinib; and pneumonitis with pralsetinib may be the result of non-RET receptor inhibition. Other side effects may be the result of inhibition of endogenous RET receptors which play a role in physiological pathways. Literature on RET physiology has demonstrated a role in the bone marrow niches and in parasympathetic neurons of the gastro-intestinal system or in the pituitary. In fact, RET is a dependence receptor in some cell types in which in the absence of ligand induces apoptosis. These parallel pathways would explain the association of Hirschsprung disease and MEN2 for some *RET* point mutations in the same patient.

## Dosing and suggested dose modifications

### Selpercatinib

The FDA-recommended dose of selpercatinib is 160 mg twice daily.^15^ If the patient weighs less than 50 kg, the recommended dose is 120 mg twice daily. No dose modification is required in case of mild or moderate renal impairment, but selpercatinib was not tested in patients with creatinine clearance (CrCl) < 30 mL/min. It is recommended to reduce the dose of selpercatinib in case of severe hepatic impairment (total bilirubin > 3 × upper limit of normal [ULN] and any Aspartate transaminase [AST] elevation), and the regular dose can be maintained in case of mild to moderate impairment (total bilirubin 1–5 × ULN and AST greater than the ULN or total bilirubin < 3 × ULN and AST within normal limits [WNL]).^15^ Table S2 shows the levels of dose modification in case of limiting adverse events.

### Pralsetinib

The FDA-recommended dose of pralsetinib is 400 mg once daily.^16^ Although selpercatinib must be adjusted by body weight, pralsetinib showed no clinically significant differences in the pharmacokinetics when tested in patients with different weights. Also, there was no clinically significant differences in pharmacokinetics with mild and moderate renal impairment (CrCl > 15 mL/min) or mild hepatic impairment (bilirubin < 1.5 × ULN and any AST level), so there is no indication for dose reduction in these scenarios. Pralsetinib was not tested in severe renal impairment (CrCl < 15 mL/min) and in moderate/severe hepatic impairment (total bilirubin > 1.5 × ULN and any AST elevation).^16^ The recommended dose reduction for adverse reactions is provided in Table S3.

## Management of common and serious adverse events

**Figure 2:**
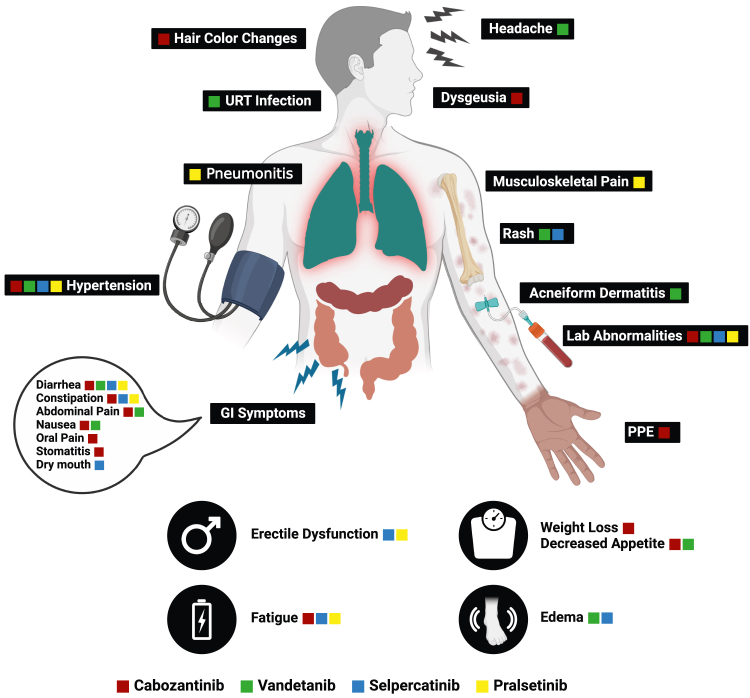
Common toxicities with different selective and non-selective RET inhibitors

Side effects due to RET inhibitors vary according to the used agent but are usually more tolerable than those caused by non-selective RET inhibitors (Figure 2).

In clinical trials of pralsetinib and selpercatinib, rates of treatment discontinuation due to treatment-related adverse events (TRAEs) were low. For example, in different cohorts of the ARROW trial, rates of discontinuation due to emerging toxicities were about 4%–7% in patients receiving pralsetinib.^9^^,^^17^^,^^18^^,^^19^ In the LIBRETTO-001 and LIBRETTO-321 trials evaluating selpercatinib, discontinuation rates were between 2% and 5%.^20^^,^^21^^,^^22^^,^^23^^,^^24^^,^^25^ Fatigue, hypertension, constipation, and diarrhea were frequent side effects common among patients treated with pralsetinib or selpercatinib, occurring in more than 25% of patients (Table 1).

**Table 1:** Common side effects occurring with selpercatinib and pralsetinib

	Selpercatinib	Pralsetinib
Most common adverse events	• fatigue • hypertension • constipation • diarrhea • nausea • edema • dry mouth • abdominal pain • rash • headache	• fatigue • hypertension • constipation • diarrhea • musculoskeletal pain
Most common grade 3 or 4 laboratory abnormalities	• decreased lymphocytes • increased ALT • increased AST • decreased sodium • decreased calcium	• decreased lymphocytes • decreased neutrophils • decreased hemoglobin • increased ALT • increased AST • decreased sodium • decreased phosphate • decreased calcium • decreased platelets • increased alkaline phosphatase

Given the high response rates with selective RET inhibitors, efforts should be made to prevent, whenever possible, or treat potential side effects to avoid compromising outcomes with treatment cessation. However, patients’ quality of life should always have priority, and physicians should balance the expected desirable outcomes with tolerability of side effects. In general, milder grades of toxicities can be managed by supplementary measures, dose reductions, or treatment interruption. Nevertheless, treatment discontinuation should be considered in cases with continued, severe, or life-threatening toxicities (Table 2).

**Table 2:** Summary of recommendations for management of selected toxicities to selective RET inhibitors

Hypersensitivity	• Withhold the drug and prescribe steroids (e.g., prednisone 1 mg/kg). • Resume after resolution of symptoms at third dose level. • Gradually increase the dose (one dose level per week) until the original dose is restored. • Continue steroids until target dose is achieved and gradually taper if symptoms remain controlled. • Permanently discontinue the drug if hypersensitivity recur.
Cutaneous toxicities	• Prescribe topical steroids (e.g., hydrocortisone 2.5% cream and/or topical clindamycin [1% gel]) for localized grade 1 or 2 toxicity. • Prescribe oral anti-histaminic in cases with associated pruritus. • Prescribe oral doxycycline (100 mg twice daily) or minocycline (100 mg twice daily) in addition to topical therapy for generalized and mildly symptomatic grade 2 rash. • Prescribe systemic steroids in generalized and severe grade ≥ 3 rash. Withhold the drug until the toxicity improves to grade ≤ 1. • Reassess patients after 2 weeks of supportive treatment. • Resume at a first level-reduced dose. Further subsequent reductions may be considered on the basis of tolerability.
Hepatotoxicity	• Regularly monitor ALT and AST every 2 weeks for the first 3 months then monthly afterward. • Withhold the drug in grade ≥ 3 events and monitor weekly until toxicity decreases to grade 1. • Resume at a reduced dose by 1 dose level for pralsetinib and 2 dose levels for selpercatinib. • Gradually increase the dose until the original dose is restored. • Permanently discontinue if grade ≥ 3 hepatotoxicity recurred.
Stomatitis	• Prescribe topical oral care, bland rinses, and topical anesthetics (e.g., 2% viscous lidocaine swish and spit). • Prescribe 2% morphine mouthwash swish and spit for severe pain. • Prescribe systemic analgesia for symptom control as appropriate. • Consider admission to hospital for fluid and diet intake in case of intense pain.
Dry mouth	• Prescribe topical mucosal lubricants or saliva substitutes, sugar-free (acidic non-erosive or non-acidic) chewing gum and acupuncture. • Prescribe oral pilocarpine or cevimeline and consider transcutaneous electrostimulation in severe cases.
Hematologic toxicities	• Obtain full blood counts prior to each treatment cycle and as clinically indicated. • Withhold treatment in patients with grade 3 or 4 toxicity. • Resume only after recovery to grade 2 or less with the possibility of dose reductions or treatment cessation. • Follow guidelines for management of chemotherapy-induced hematological complications. • Use supportive measures including hematopoietic growth factors whenever appropriate.
Hemorrhagic events	• Withhold the drug in grade ≥ 2 toxicities. • Resume only after full recovery to grade 0 or 1. • Use supportive measures including possible blood transfusions as appropriate. • Permanently discontinue in patients with severe or life-threatening hemorrhage.
QT interval prolongation	• Monitor and correct QT interval, electrolytes, and TSH before starting treatment and periodically while on therapy. • Withhold the drug in grade ≥ 3 toxicities. • Resume only after full recovery to grade 0 or 1 at a reduced dose. • Permanently discontinue in cases with grade 4 events.
Hypertension	• Do not start treatment with RET inhibitors in patients with uncontrolled hypertension. • Regularly monitor hypertension preferably for 1 week then monthly afterward. • Add or optimize hypertensive medications as appropriate in patients with treatment-emergent hypertension. • Withhold the drug in patients with persistent grade 3 or 4 toxicities.
Wound healing	• Withhold selpercatinib 7 days and pralsetinib 5 days before any planned surgery. • Resume after a minimum hold of 2 weeks post-surgery.
Pneumonitis	• Withhold the drug in patients with any grade ILD/pneumonitis. • Prescribe prednisone 1–2 mg/kg/day and taper over 4–6 weeks. • Prescribe empiric antibiotics if infection remains in the differential diagnosis after workup. • Resume treatment after full recovery with appropriate dose reductions. • Permanently discontinue in cases with grade 3 or 4 events and if toxicity recurred for four times despite increasing dose reductions. • Prescribe methylprednisolone i.v. 1–2 mg/kg/day for patients with grade 3 or 4 events. Consider adding immunosuppressive agent (e.g., infliximab, mycophenolate mofetil i.v., IVIG, or cyclophosphamide) if no improvement occurred after 48 hours.
Edema and chylous effusions	• Use drainage for immediate symptoms relief in symptomatic effusions. • Include a high-protein and low-fat diet with medium-chain triglycerides, reducing the ingestion of long-chain triglycerides. • Consider orlistat or octreotide for controlling the volume of effusion.
Fatigue	• Rule out other causes (e.g., anemia, hypothyroidism, malnutrition). • Consider short-term dexamethasone or methylprednisolone. • Withhold the drug in grade ≥ 3 and resume with a dose reduction.
Tumor lysis syndrome	• Prophylaxis with hydration, monitoring, and allopurinol or rasburicase in high-risk patients. • Prescribe i.v. fluid with 2–3 L/m2 of isotonic saline. • Prescribe allopurinol or rasburicase. • Correct electrolytes abnormalities and consider dialysis if refractory. • Withhold the drug until TLS is resolved and resume with dose reduction.
Reproduction	• Discuss the potential for causing infertility with the patient before starting. • Check pregnancy status in women of reproductive potential prior to initiating therapy. • For selpercatinib, prescribe effective contraception during therapy and up to one week after. For pralsetinib, prescribe non-hormonal contraception during treatment and for 2 weeks after. • Counsel the patient to abstain from breast feeding during therapy and up to 1 week after.
Hypothyroidism	• Lower T3 levels, although normal T4. • Thyroidectomized patients may need supplementation with liothyronine.

It is currently unclear if one of the two drugs would be superior to the other in certain clinical scenarios in which patients had baseline toxicity that can be possibly exacerbated by drug-specific toxicity. Similarly, it is not certain if the reintroduction of another drug after withdrawal of the original RET inhibitor because of toxicity would be tolerable. This might be plausible in cases in which non-overlapping toxicities are observed and will remain an area of interest for future studies. It is important to realize that although most of the side effects occur across all disease groups, certain associations were also reported in clinical trials. For example, tumor lysis syndrome is reported in higher grade and bulky disease thyroid cancer and has not been seen in lung cancer. Thyroid hormone fluctuations are seen primarily in patients with thyroid cancer. Moreover, lung cancer patients who come off immunotherapy have reported more hypersensitivity reactions than any other tumor type. It would be important to follow real-world data to see if any of the side effects are more pronounced in specific tumor types.

### Hypersensitivity reactions

Selpercatinib showed hypersensitivity of any grade in 4.3% of patients treated in clinical trials.^9^^,^^21^^,^^26^ Most of the events occurred between the first and second week after starting the treatment. Symptoms included fever, rash, hypotension, tachycardia, and arthralgia/myalgia, and the laboratory workup showed thrombocytopenia, transaminitis, and creatinine elevation, with a more frequent incidence among patients harboring *RET* fusions. Among the patients presenting with hypersensitivity, 77% had received immune checkpoint inhibitors prior to treatment with selpercatinib.

Pralsetinib trials did not report systemic hypersensitivity syndrome, and the only hypersensitivity symptom reported was rash without grade 3 events.^9^^,^^17^

Regardless of grade, the recommendation for hypersensitivity is to withhold the drug and prescribe steroids. The RET inhibitor may be resumed once symptoms completely resolve and restarted at the maximally recommended dose reduction (e.g., the third dose level) (Tables S2 and S3). In each week, the dose can be increased until achieving the original dose used by the patient before the hypersensitivity event. Steroids must be continued until the target dose and, if the patient continues with controlled symptoms, it can be tapered gradually. In case of recurrence of the hypersensitivity reaction, it is recommended to permanently discontinue the RET inhibitor.^27^

### Cutaneous toxicities

Tyrosine kinase inhibitors (TKIs) are known to cause dermatologic toxicities because of the inhibition of the VEGFR or EGFR pathway.^28^^,^^29^^,^^30^^,^^31^ Selective RET inhibitors have a less pronounced effect in the other kinases, via sparing the VEGFR.^32^ For example, with selpercatinib, skin rash occurred in 27% of patients, with only 0.7% (one patient) having grade 3 or higher toxicity.^19^^,^^20^ Similarly, 24% of pralsetinib-treated patients had rash, with no grade 3 toxicity.^19^^,^^27^ The types of rashes reported were erythematous, macular, maculopapular, morbilliform, and pruritic.

As there is no robust literature for selective RET inhibitors, and considering that they probably share the same mechanism of toxicity with other TKIs, we recommend that cutaneous toxicities of selective RET inhibitors can be managed in an analogous way to the non-selective inhibitors.

In case of non-limiting and localized grade 1 or 2 toxicities, the patient may receive topical corticosteroids. If rash presents with associated pruritis, an oral antihistamine can be prescribed.^33^ For generalized and mild symptomatic grade 2 rash, oral doxycycline or minocycline can be added.

When the rash presents as a generalized and severe symptom (grade ≥ 3), systemic steroids may be required to manage the rash. In this case, the RET inhibitor must be withheld until the toxicity improves to grade ≤ 1.

All patients with a new onset rash, regardless of the grade of severity, must be reassessed in 2 weeks after starting supportive treatment. Upon resuming the RET inhibitor, it may be necessary to reduce the dose by 1 dose level (Tables S2 and S3) and consider subsequent dose reductions according to the patient tolerability.^28^

### Transaminase elevation

Alanine transaminase (ALT) and AST elevations occurred in approximately half of the patients receiving RET inhibitors (51% for AST and 45% for ALT with selpercatinib, 69% for AST and 46% of ALT with pralsetinib), although grades ≥ 3 occurred in fewer than 10% (8% for AST and 9% for ALT with selpercatinib, 5.4% for AST and 6% for ALT with pralsetinib). Timing to transaminase elevation may be variable, with some patients experiencing this in less than one week of treatment while others present with hepatotoxicity after one year of treatment.^10^^,^^13^^,^^19^^,^^27^

It is recommended that ALT and AST be monitored every 2 weeks for the first 3 months while the patient is receiving a RET inhibitor. In subsequent months, if no hepatotoxicity occurs, it is reasonable to assess ALT/AST monthly or as clinically indicated.^10^^,^^13^^,^^19^^,^^27^

According to the guidelines of the European Association for the Study of the Liver, for any grade of transaminases elevation, a thorough personal/social history of the patient should be sought about the use of alcohol, drugs, herbal and dietary supplements, and medications known to cause hepatotoxicity.^34^ It is also reasonable to order viral hepatitis tests (hepatitis A virus [HAV] IgM, hepatitis B surface antigen [HBsAg], hepatitis C virus [HCV] RNA, hepatitis E virus [HEV] IgM, and herpes simplex virus [HSV]) and consider an abdominal ultrasound with Doppler.^35^ Possible risk factors such as age, family history, concomitant auto-immune disease, and components of metabolic syndrome must be assessed to rule out other etiologies. It is recommended to consider a hepatologist evaluation in case the toxicity becomes severe, prolonged, or unresponsive to the initial management.^34^^,^^35^

For patients with ALT/AST elevation grades 1 and 2, there is no recommendation for suspending the RET inhibitor, and this decision may be based on the physician’s discretion. In cases with grade > 3, the RET inhibitor must be withheld, and ALT/AST must be monitored once a week, until it improves to grade 1. If the treatment is resumed, pralsetinib must be prescribed at a reduction of 1 dose level (Table S3). If there is a recurrence of the hepatotoxicity at grade ≥ 3, the RET inhibitor must be discontinued definitively. For selpercatinib, it is recommended that it be resumed with a reduction of 2 dose levels (Table S2) and that the dose may be increased gradually until achieving the original dose (taken prior to hepatotoxicity), within at least 4 weeks from the restarting date. On the basis of the severity of the event, permanent discontinuation of the treatment may be required.^8^^,^^36^

### Stomatitis and dry mouth

The mechanism of stomatitis secondary to targeted therapy is not completely known, but it is possibly related to some VEGFR inhibition, in a pathophysiology that is different from cytotoxic chemotherapies.^37^ Selective RET inhibitors cause a lower incidence of stomatitis compared with cabozantinib and vandetanib, so the clinical relevance and drug discontinuation because of stomatitis are less common with selpercatinib and pralsetinib. There are no reports of stomatitis in trials including patients who received selpercatinib.^21^^,^^22^ Pralsetinib was found to cause stomatitis in 17% of the patients with one case of a grade 3 event.^9^^,^^17^^,^^19^

As the literature lacks information about the management of stomatitis secondary to targeted therapy, we suggest a similar approach to that currently used for stomatitis attributed to cytotoxic therapy, according to our institutional experience. Also, extrapolating from oncology guidelines about general cancer treatment’s adverse events, prophylaxis with adequate oral hygiene and diet must be reinforced to all patients,^38^ and topical oral care plus systemic analgesia can be used for symptom control.^39^ Options are bland rinses and topical anesthetics, such as 2% viscous lidocaine swish and spit. In case of severe pain, 2% morphine mouthwash swish and spit is a reasonable option. In case of intense pain, fluid intake and diet must be assessed with consideration of hospital admission for symptom management.^39^

Xerostomia is a common side effect of antineoplastic treatments, reported in 39% of patients on selpercatinib and 16%–17% of patients on pralsetinib.^10^^,^^13^ Considering the relatively low incidence of severe cases, we do not suggest preventive measures with selective RET inhibitors. This may be more pronounced in patients with thyroid cancer with histories of radioactive iodine and head and neck irradiation.

Although there is no specific recommendation for xerostomia caused by targeted therapies, the general guidance from International Society of Oral Oncology (ISOO)/Multinational Association of Supportive Care in Cancer (MASCC)/American Society of Clinical Oncology (ASCO) suggest topical mucosal lubricants or saliva substitutes, sugar-free (acidic non-erosive or non-acidic) chewing gum, and acupuncture. For severe cases, we suggest a similar approach to patients undergoing head and neck radiation, with oral pilocarpine or cevimeline, and transcutaneous electrostimulation.^40^

### Hematologic toxicity

Treatment-induced decrease in blood counts have been consistently reported in studies of both selpercatinib and pralsetinib.^9^^,^^17^^,^^18^^,^^19^^,^^21^^,^^24^ In fact, treatment-related lymphopenia, neutropenia, and anemia were among the most commonly encountered grade 3 or 4 toxicities in the ARROW trial of pralsetinib regardless of tumor type.^9^^,^^17^^,^^18^^,^^19^ Furthermore, neutropenia was the most common toxicity leading to dose reduction or treatment interruption in updated analysis of the ARROW trial.^19^ Trials of selpercatinib showed a relatively lower frequency of hematological adverse events compared with pralsetinib, but they were not infrequent. For example, in the LIBRETTO-001 trial, treatment-related thrombocytopenia occurred in 14.6% of patients receiving selpercatinib.^21^ Thrombocytopenia led to dose reductions in 5.2% of patients in the LIBRETTO-321 study.^24^ As hematological toxicities are primarily laboratory findings, most cases can be asymptomatic. However, complications can occur because of low blood counts, including but not limited to life-threatening infections and serious bleeding.

Given the negative outcomes such toxicities can lead to, because of either their direct impact or frequent alterations in treatment schedules, vigilance should be practiced when prescribing RET inhibitors. We suggest full blood counts should be obtained prior to each treatment cycle and as clinically indicated. Treatment should be stopped in patients developing grade 3 or 4 events and resumed only after recovery to grade 2 or less, with the possibility of dose reductions or treatment cessation. Patients should be counseled about the risk for hematological toxicity and made aware of symptoms and signs suggestive of complications due to low blood counts. This counseling may include recommendations to avoid situations when they could be exposed to infections. Throughout treatment, guidelines for management of chemotherapy-induced hematological toxicities and complications should be followed. Use of supportive measures including hematopoietic growth factors may be needed.

### Hemorrhagic events

Hemorrhagic events, not necessarily related to thrombocytopenia, may occur in some patients who are receiving selpercatinib or pralsetinib and may be life threatening. In the LIBERETTO-001 trial, nearly 2% of patients were reported to have grade 3 or 4 hemorrhage.^8^^,^^15^ Similarly, in the ARROW trial, grade 3 or 4 bleeding events occurred in 2.5% of patients on pralsetinib.^8^^,^^16^ In addition to either local or systemic bleeding, patients may also present with bleeding-related complications, including reduction in their hemoglobin.

As with other drugs with this risk, we recommend that patients treated with selpercatinib or pralsetinib should be informed of the increased risk for bleeding complications and report any new or worsening events to their treating physician. RET inhibitors should be permanently discontinued in patients who develop severe or life-threatening hemorrhage after treatment initiation, according to prescribing information. In milder presentations, treatment may be withheld until full recovery to grade 0 or 1. Supportive management including possible blood transfusions may be needed in some patients with serious bleeding events.

### QT interval prolongation

QT interval prolongation is a well-documented side effect of TKIs, related to repolarization abnormalities.^8^^,^^41^ Notably, this toxicity is exclusively limited to selpercatinib alone. Cardioelectrophysiology studies demonstrated that the increase in corrected QT (QTc) interval was concentration dependent, with the largest mean increase in QTc interval at 10.6 ms at the mean steady-state maximum concentration (Cmax) after the administration of 160 mg twice daily. In the LIBRETTO-001 trial with selpercatinib, grade ≥ 3 TRAEs of QT interval prolongation were reported in 4% of patients with dose interruptions and reductions in >2% of patients. In the ARROW study, no effect of pralsetinib on QT interval was observed.^9^

It is important to ask patients about symptoms of dizziness, syncope, palpitations, and loss of consciousness and to screen for concomitant medications that are associated with QTc interval prolongation, a history of long-QT syndrome, symptomatic bradyarrhythmias, severe or uncontrolled heart failure, and diarrhea. We also suggest that monitoring and correction of QT interval, electrolytes, and thyroid-stimulating hormone (TSH) at baseline prior to initiation and periodically during treatment are vital.

In the event of grade 3 QT interval prolongation, selpercatinib must be held until recovery to baseline or grade 0 or 1 and then resumed at a reduced dose, as recommended in the manufacturer’s prescribing information (Table S2).^8^^,^^41^ In the case of a grade 4 event, selpercatinib must be discontinued permanently.

We also recommend optimizing thyroid hormone, calcium, phosphorus, and magnesium levels, which could all affect the QT interval.

### Hypertension

Hypertension is a common toxicity that has been frequently reported with different kinase inhibitors, probably due to cross-inhibition of VEGFR receptors in normal blood vessels.^41^ Despite the RET selectivity of selpercatinib and pralsetinib, both drugs have been associated with an increased risk for hypertensive events in patients receiving either agent especially at the highest doses and with chronic dosing.^8^^,^^15^,^16^^,^^36^^,^ Significant proportions of patients have experienced blood pressure elevation, making hypertension one of the most common adverse reactions associated with selpercatinib and pralsetinib use.^9^^,^^17^^,^^18^^,^^19^^,^^21^ Hypertension was one of the most common grade 3/4 toxicities in the clinical trials of selpercatinib and pralsetinib^17^^,^^18^^,^^19^^,^^22^ and led to dose interruptions or subsequent dose reductions in some patients.

According to prescribing information, treatment with RET inhibitors should not be initiated in patients with uncontrolled hypertension. Hypertension management by a cardiologist or other experienced provider should be considered in such cases for blood pressure optimization. Blood pressure should be frequently monitored in patients beginning at baseline, after 1 week of therapy, then monthly thereafter. Treatment-emergent hypertension can be managed with addition or optimization of anti-hypertensive medications as appropriate. Drugs should be stopped in patients presenting with persistent grade 3 or 4 toxicities. Dose reduction or treatment cessation may be indicated in some patients depending on severity and response to anti-hypertensives.^15^^,^^16^ Patients should be aware of symptoms and signs that are indicative of complications and should report any elevated blood pressure readings to their physician.

### Wound-healing issues

Impaired wound healing can occur in patients who receive selpercatinib or pralsetinib by inhibiting the VEGF1–3 signaling pathway. In anticipation of a planned surgical procedure, selpercatinib should be held 5–7 days and pralsetinib should be held for 5 days prior to the procedure. Resumption of both drugs should be considered after a minimum hold of 2 weeks following major surgery.^15^^,^^36^ A recently published case series did not demonstrate impaired wound healing or other surgery-associated complications with neoadjuvant selpercatinib followed by surgery for locoregionally advanced *RET*-altered thyroid cancer patients.^42^ An ongoing phase II clinical trial is studying the efficacy and safety of selpercatinib prior to surgery for locoregionally advanced *RET*-altered thyroid cancer (NCT04759911).^43^

### Pneumonitis

Pneumonitis and drug-induced interstitial lung disease (ILD) can occur with RET inhibitors, especially with pralsetinib. In general, they have been reported in about 10% of pralsetinib cases, including 2.7% grades 3 and 4, 0.5% including fatal reactions, and less than 2% with selpercatinib use.^18^^,^^21^

The etiopathogenetic mechanisms of pneumonitis and drug-induced ILD in targeted molecular therapies are not well known.^44^ As portions of the investigational trials were conducted during the COVID-19 pandemic, it is unknown if a history of COVID-19 infection can potentiate pneumonitis.

According to the American Thoracic Society/European Respiratory Society, idiopathic interstitial pneumonias is classified into three groups, considering clinical and pathological features: major idiopathic interstitial pneumonias, rare idiopathic interstitial pneumonias and unclassified idiopathic interstitial pneumonias.^45^

The prompt diagnosis and evaluation of the differential diagnosis are crucial to the prognosis because of severe adverse events (SAEs). In that case, toxicity management must start once the diagnosis is made. It is mandatory to exclude COVID-19 infection and other lung pathologies.^46^ Clinical examination and patient monitoring are mandatory. Laboratory tests can be unspecific, but excluding rheumatological diseases, myositis, is important. Respiratory cultures should be collected empirically. Also, high-resolution computed tomography (HRCT) of the chest is mandatory. Cardiac evaluation should be performed to identify concomitant cardiac disease. Additional tests/procedures including electrocardiography (ECG), brain natriuretic peptide (BNP), and, if needed, echocardiography may further help clarify or exclude cardiac etiologies. Pulmonary function tests may be necessary in case diagnosis is still unclear. Also, bronchoalveolar lavage is important if this is the first incident of ILD/pneumonitis; if after all exams the etiology is still unknown, a lung biopsy may be needed to define the diagnosis.^47^

If ILD/pneumonitis grade 1 or 2 occurs, pralsetinib should be held until resolution. Treatment should be resumed with a dose reduction (Table S3). If ILD/pneumonitis grade 3 or 4 occurs, pralsetinib must be permanently discontinued.^18^ Regarding ILD/pneumonitis related to selpercatinib, the reported incidence is less than 2%. In case of occurrence, a dose reduction after treatment has been held should follow the instructions according to Table S2.

### Edema and chylous effusions

A common side effect of selective RET inhibitors is the occurrence or worsening of edema, including peripheral, facial, eyelid, and other generalized presentations (as pleural/pericardial effusions and ascites). This occurred in 33% of patients taking selpercatinib (with one grade 3 event) and 20%–29% with pralsetinib.^10^^,^^12^^,^^13^ Although this is usually not severe or life threatening, it may be a factor responsible for impairing the quality of life of patients. As in other types of chronic edema, we suggest managing it with postural and compressive measurements for mild peripheral edema. If the patient presents with significant thoracic effusion or ascites, it is important to consider thoracentesis or paracentesis, with the intent not only to alleviate symptoms but also to rule out chylous ascites and chylothorax.

Chylous ascites and chylothorax may occur secondary to some multi-kinase and selective RET inhibitors. We consider its recognition important because some patients could be mistakenly taken off the targeted treatment because of the physician’s interpretation of the event as being related to progression of disease. It also requires a specific treatment that is different from a malignant pleural effusion or ascites.

The association between chylous effusions and selective RET inhibitors has been described in some case reports and recently a systematic dataset of patients with *RET*-altered tumors described an incidence rate of 7% among patients receiving selpercatinib.^10^^,^^48^ The mechanism of this entity is still unclear, and its management is not established. We suggest following the same guidelines used for chylous ascites/chylothorax from other etiologies, as there is no robust literature.

The diagnosis is confirmed with the analysis of the triglyceride level in fluid. Patients with symptomatic effusions benefit from drainage for immediate symptom relief. The long-term management must include a high-protein and low-fat diet with medium-chain triglycerides, reducing the ingestion of long-chain triglycerides. Orlistat and octreotide are pharmacologic options to help control the volume of effusion.^49^ A dose reduction did not promote benefit in the published dataset with 15 patients, although the investigators evaluated a small number of patients.^10^^,^^48^

### Erectile dysfunction

Erectile dysfunction (ED) has been reported clinically in patients on selective RET inhibitors, according to our institutional experience. After checking for testosterone levels and optimizing thyroid hormone levels, we suggest consulting urology for ruling out all other causes of ED. Sildenafil and tadalafil have been effectively used in patients to alleviate ED at our institution.

### Withdrawal effects

Although not common, we have seen a few patients suffer from RET inhibitor withdrawal, as manifested by myalgia, flulike symptoms, fatigue, flushing, sweating, headache, and nausea. In such patients, it is recommended to slowly taper the dose of the drugs over a period of 1 week to alleviate the symptoms of withdrawal. Acetaminophen and non-steroidal anti-inflammatory drugs such as ibuprofen can help with non-specific myalgias in our experience. Hydration has also helped patients as well.

### Fatigue

Fatigue is one of the most common adverse events that occur in patients taking selective RET inhibitors. The proposed mechanism is related to the previous angiogenic VEGF or TKI treatments.^50^^,^^51^

If the patient develops fatigue grade 3, treatment should be held and resumed with the first dose reduction after recovery to grade 2. If subsequent reductions are needed until the minimal dose, treatment needs to be discontinued (Tables S2 and S3).^15^^,^^36^

In the LIBRETTO-001 trial, fatigue (including malaise and asthenia) occurred in 46% of all grades of adverse events and 3.1% of grade 3 or 4 SAE patients using selpercatinib. Dose reduction in more than 2% of patients occurred because of toxicity, including fatigue. Permanent discontinuation was seen in 0.6% of patients.^8^^,^^15^ In the ARROW trial, fatigue (including asthenia) occurred in 35% of all adverse event grades and 2.3% of grade 3 or 4 SAE patients using pralsetinib. There was no permanent dose discontinuation because of fatigue among the 15% of patients who permanently discontinued the treatment.^36^

Cancer-related fatigue (CRF), thyroid dysfunction, anemia, malnutrition, and depression are among differential diagnoses. There is no literature about the treatment of fatigue related to RET inhibitor. We suggest holding RET-inhibiting treatment and reducing the dose in severe cases.

According to European Society for Medical Oncology (ESMO) and ASCO guidelines, nonpharmacological intervention is essential for screening, assessment, education, and appropriate treatment of fatigue, on the basis of the patient’s needs.^52^^,^^53^ Pharmacological intervention is needed when patients score CRF 4 or more (1–10 CRF scale). The strongest recommendation is class IIB and recommends short-term dexamethasone or methylprednisolone for the control of CRF in metastatic cancer patients.^53^ Also, cognitive behavior therapy, mind-body intervention, and some psychostimulants can help in the management of fatigue,^53^ and there are no clinically relevant drug interactions between these medications and the selective RET inhibitor with the exception of moderate inducers of CYP3A4.

### Tumor Lysis Syndrome

Tumor lysis syndrome is characterized by various electrolyte abnormalities, including hyperkalemia, hyperphosphatemia, hyperuricemia, hypocalcemia, and metabolic acidosis. The mechanism of action is cancer cell death, caused primarily by the treatment agent.^54^

If the patient has suspected tumor lysis syndrome, treatment needs to be held and resumed after recovery of tumor lysis syndrome, with the first dose reduction (Tables S2 and S3) and subsequent dose reductions. Treatment should be discontinued if recurrent grade 4 adverse event.^8^^,^^36^

Approximately 0.6% of patients with medullary thyroid carcinoma (MTC) receiving selpercatinib had tumor lysis syndrome. There were also case reports of tumor lysis syndrome among patients with MTC in use of the pralsetinib.^8^^,^^15^ Risk factors are high tumor burden, renal dysfunction, dehydration, and fast-growing tumor. Also baseline uric acid, potassium, and or phosphate greater than the ULN should be highly considered risk factors.

Although most solid tumors have a low risk for developing tumor lysis syndrome, we should consider those with intermediate-risk disease (containing at least one or more risk factors such as renal dysfunction/renal involvement, or uric acid, potassium, and or phosphate greater than the ULN).^54^

Prophylaxis with hydration, monitoring, and allopurinol or rasburicase should be considered in these scenarios. If clinically significant, it needs to be treated immediately. It is important to note that rasburicase is contraindicated in patients with glucose-6-phosphate dehydrogenase (G6PD) deficiency and should be replaced by allopurinol.^15^

Aggressive hydration should be initially accomplished through administration of intravenous (i.v.) fluid with 2–3 L/m^2^ isotonic saline, and urinary output should be monitored and maintained at 80–100 mL/m^2^/hour.^54^

Hypouricemic agents are allopurinol 100 mg/m^2^ orally every eight hours (maximum of 800 mg/day) or 200–400 mg/m^2^ i.v. daily (maximum of 600 mg/day) if the patient cannot take oral medications.^54^ Rasburicase is another option and may be administered as weight-based daily dosing for up to 7 days with the dose based on TLS risk: 0.2 μg/kg/day for high risk, 0.15 μg/kg/day for intermediate risk, and 0.1 μg/kg/day for low risk.^54^ Single, fixed-dose administration strategies of rasburicase have also been evaluated, with doses ranging from 3 to 7.5 mg and repeated doses used on the basis of uric acid levels.^55^

Febuxostat is a nonpurine xanthine oxidase inhibitor that is safe in renal impairment and can be an alternative for cases of resistance or intolerance to allopurinol when rasburicase is contraindicated.^56^ The dose is 40–60 mg/day oral^57^ or 120 mg/day oral,^58^ starting one or two days before targeted treatment (in the context of prophylaxis) and continuing up to 14 days until laboratory normalization and the risk for TLS has subsided.

The treatment involves correcting the electrolyte abnormalities that remain over the prophylaxis. Renal replacement therapy is indicated if conservative treatment is not effective.^59^

### Reproduction

Selpercatinib and pralsetinib have demonstrated teratogenic effects in animal reproduction studies and hence may cause fetal harm when administered in pregnancy. Therefore, it is paramount to check pregnancy status in women of reproductive potential prior to initiating therapy. Malformations and embryo lethality were seen in pregnant rat models with selpercatinib at clinical dose of 160 mg twice daily and pralsetinib at clinical dose of 400 mg once daily. For selpercatinib, both male and female partners should be advised to use effective contraception during therapy and up to one week after the final dose. For pralsetinib, female partners of reproductive potential should use non-hormonal contraception during treatment and for 2 weeks after the final dose, while male partners should use effective contraception during therapy and up to one week after the final dose.^8^^,^^36^

In lactation, because of insufficient data on the presence of selpercatinib or pralsetinib or its metabolites in human milk or on their effects on the breastfed child or on milk production, patients should be counseled to abstain from breast feeding during therapy and up to 1 week after the final dose. Animal studies suggest that selpercatinib and pralsetinib may impair fertility.^8^^,^^36^

### Hypothyroidism

A recent publication described the hypothesis of an off-target effect of selpercatinib on T3 production, leading to hypothyroidism with lower T3 levels regardless of the proper levothyroxine replacement.^60^ The mechanism appears to be related to the inhibition of the type 2 iodothyronine deidodinase (D2). Deiodinase type 2 (DIO2) is the key enzyme converting T4 in active T3 in target organs (such as pituitary, brain, skin, etc.). In this study, treatment with selpercatinib was shown to inhibit D2-mediated T3 production. This was more marked in patients with athyreotic status where there was a need to add liothyroxine to levothyroxine in order to maintain normal T3 level and alleviate hypothyroid symptoms. In patients with functioning thyroid, less decrease was observed in serum T3 levels, which is likely related to the fact that any reduction in peripheral conversion of T4 to T3 was offset by increased TSH-stimulated thyroid T3 production. In patients with NSCLC, the reduction in T3 was also noted, but the fact that they still had a functioning thyroid probably compensated the production of the hormone. In thyroidectomized patients, the supplementation with liothyronine was needed in order to achieve a normal T3 level, thus controlling some symptoms of hypothyroidism.^60^ This study did not test if pralsetinib also disturbs the production of T3.

## Common drug-drug interactions and dose modifications for cytochrome P450 family 3 subfamily A inhibitors

The patient’s current medication use including prescription and over-the-counter (OTC) medications, herbal supplements, and multivitamins must be reviewed for potential drug-drug interactions.

### Selpercatinib

Combination of selpercatinib with a strong or moderate cytochrome P450 family 3 subfamily A (CYP3A4) inhibitor increases selpercatinib plasma concentrations, which may increase the risk for QTc interval prolongation, making serial ECG monitoring necessary. Table S4 shows the levels of dose modification when concurrent use of strong and moderate CYP3A4 inhibitors are needed.

Strong, moderate, and weak CYP3A4 inducers are noted to decrease selpercatinib levels, and hence attention to drug-drug interactions is paramount in concurrent use with selpercatinib. An exhaustive list of drug interactions beyond the scope of this review can be found in a drug information database such as Lexicomp.^61^

### Pralsetinib

Notably, no interactions with mild CYP3A4 inducers, proton-pump inhibitors (PPIs), H2 receptor antagonists, or antacids are seen with pralsetinib. However, concurrent use with known combined P-glycoprotein (P-gp) and strong CYP3A4 inhibitors demonstrates increase plasma pralsetinib concentration. If concurrent P-gp or strong CYP3A4 inhibitor is needed, reduction in the current dose of pralsetinib is recommended in Table S5.

Concurrent use of pralsetinib with strong CYP3A4 inducers can lead to decrease plasma concentrations of pralsetinib. Once the inducing agent has been discontinued for at least 14 days, pralsetinib can be resumed at the dose taken prior to initiating the strong CYP3A4 inducer. However, if concomitant use of a strong CYP3A inducer is required, one can increase the starting dose of pralsetinib to double the current dose starting on day 7 of coadministration of pralsetinib with the strong CYP3A4 inducer. An exhaustive list of drug interactions beyond the scope of this review can be found in Lexicomp.^62^

## Special cases

### Pediatric, adolescent, and young adult considerations

Selpercatinib and pralsetinib are safe for adolescents older than 12 years old, but there are no safety data for children younger than this age. The indications for which it has been approved for adolescents are medullary thyroid cancer and *RET*-fusion-positive thyroid cancer. There is no support in the labels for other neoplasms with RET alterations.^10^^,^^12^^,^^13^^,^^36^

Data on toxicity in juvenile animals showed irreversible decreased femur length and reduced bone mineral density due to growth plate changes. There are also data reporting tooth abnormalities.^8^^,^^36^

Additionally, there is a concern regarding sexual maturity and fertility issues secondary to the use of selpercatinib at young ages, according to animal model experiments.

A key recommendation is to monitor growth plates in adolescent patients who still show open growth plates in the baseline radiography and consider interrupting or discontinuing therapy according to the severeness of the alterations.

### Geriatric use

There are no guidelines for targeted therapy in older patients (≥65 years) according to ASCO guidelines for geriatric oncology.^63^ Also, the French Society of Geriatric Oncology (SoFOG) corroborates the same, with a cutoff age of ≥70 years, in a systematic review of targeted therapy for older patients with NSCLC.^64^ SoFOG guidelines suggest using targeted therapy monotherapy and assessing comorbidities before starting the agent without a specific reference to RET inhibition.

A risk factor that can decrease the outcome of treatment in older patients is a decline in life-space mobility, which is associated with lower pre-treatment performance status, dependence for activities of daily living (ADLs), abnormal Montreal Cognitive Assessment score, lower quality of life, and higher morning fatigue.^65^

The management of toxicity treatment with dose delay and dose reduction is a challenge for oncologists who are concerned about affecting the efficacy.^65^ More studies in older patients are crucial to give more guidance on the basis of real-world data, and the design of more clinical trials that allow older patients enrollment and adapt to their peculiarities is highly recommended.

### Concomitant acid-reducing agents

The concomitant use of PPI and selpercatinib may lead to a reduced serum concentration of selpercatinib if given during fasting, as the usual posology. Hence, PPIs, H2 receptor antagonists, and antacids should be avoided. If patient needs acid-reducing agents, H2 receptor antagonists are the preferred choice. In cases in which the use of PPIs is mandatory, we recommend taking the selpercatinib in a different time of the day and after food, considering the information from pharmacokinetics studies.^8^ Selpercatinib must be given 2 h before or 10 h after administration of the H2 receptor antagonist. In the case of antacid use, selpercatinib should be given 2 h before or 2 h after antacid administration.

Pralsetinib has no significant alterations of the serum concentration when coadministered with gastric-acid-reducing agents.^36^

### Drug interactions with COVID-19 medications

The COVID-19 pandemic has altered the practice of oncology. The main challenge with co-managing COVID-19 and *RET*-altered cancers is the potential for drug-drug interactions between treatment regimens of both diseases. For example, ritonavir, used in ritonavir-boosted nirmatrelvir antiviral therapy, is a strong inhibitor of CYP3A4, which is involved in metabolism of both selpercatinib and pralsetinib.^66^^,^^67^ Concomitant use can increase the risk for TRAEs, so selpercatinib and pralsetinib should be temporarily discontinued during antiviral treatment. Another FDA-approved drug to treat COVID-19 is molnupiravir, for which preliminary data on pharmacokinetics (PK) and metabolism suggest that the risk for drug interaction with other drugs is low.^68^

### Conclusions

Selective RET inhibitors, including selpercatinib and pralsetinib, have led to a paradigm change in treatment of *RET*-altered cancers. Their toxicity and safety profile are quite tolerable, but awareness of possible side effects and approaches to management is critical in the precision oncology era. Patients’ involvement and multidisciplinary discussions might help prevent and manage toxicities and therefore should be part of standard-of-care practice.

## Supplemental information

Document S1. Tables S1–S5

Document S2. Article plus supplemental information
