# Supplementary material for: Strategies for mitigating adverse events related to selective RET inhibitors in patients with RET-altered cancers
Source: Cell Rep Med. 2023 Dec 19;4(12):101332. doi: 10.1016/j.xcrm.2023.101332 (PMC10772460; doi:10.1016/j.xcrm.2023.101332)
Supplement: Document S1. Tables S1–S5 [file mmc1.pdf]

Cell Reports Medicine, Volume *4*

Supplemental information

Strategies for mitigating adverse events related to selective RET inhibitors in patients with RET-altered cancers

Mirella Nardo, Mohamed A. Gouda, Blessie E. Nelson, Carmelia M.N. Barreto, J. Hoyt Slade, Anna Poullard, Mark Zafereo, Mimi I. Hu, Maria E. Cabanillas, and Vivek Subbiah

SUPPLEMENT

Supplementary Table 1. ORR among the different RET inhibitors

Reference	Phase	RET inhibitor	Disease	Response rate for RET alterations (%)
Schlumberger et al., 2017[1]	III	cabozantinib	RET-mutant MTC	28 (34% if RET M918T mutation)
Drilon et al., 2016[2]	II	cabozantinib	RET-fusion NSCLC	28
Wells et al., 2012[3]	III	vandetanib	RET-mutant MTC*	30.9-51.8
Wirth et al., 2020/2022[4]	I/II	selpercatinib	RET-mutant MTC**	73 (69% if previous non-selective RET inhibitor)
Drilon et al., 20202022[5]	I/II	selpercatinib	RET-fusion NSCLC	85 (64% if previous platinum treatment)
Subbiah et al., 2022[6] (LIBRETTO-001)	I/II	selpercatinib	RET-fusion pan-tumor (except MTC and NSCLC)	43.9
Subbiah et al., 2021[7] (cohort from ARROW trial)	I/II	pralsetinib	RET-mutant MTC or RET-fusion thyroid cancer	71 (89% if RET-fusion thyroid cancer; 60% of ORR in second line)
Griensinger et al., 2022[8] (cohort from ARROW trial)	I/II	pralsetinib	RET-fusion NSCLC	72
Subbiah et al., 2022[9] (ARROW trial	I/II	pralsetinib	RET-altered pan-tumor (except MTC and NSCLC)	57

*Patients with MEN2A (type 2A multiple endocrine neoplasia), MEN2B (type 2B multiple endocrine neoplasia), or FMTC (familial medullary thyroid cancer) and a germline RET mutation were eligible.

**Mutations non-specified in the inclusion criteria

Supplementary Table 2. Selpercatinib suggested dose modification

**Dose reduction**	**Less than 50 Kg**	**$\geq$ 50 Kg**
First	80 mg orally twice daily	120 mg orally twice daily
Second	40 mg orally twice daily	80 mg orally twice daily
Third	40 mg orally once daily	40 mg orally twice daily

Supplementary Table 3. Pralsetinib suggested dose modification

**Dose reduction**	**All patients**
First	300 mg orally once daily
Second	200 mg orally once daily
Third	100 mg orally once daily

Supplementary Table 4: Selpercatinib Dose Modifications when Combined with Moderate and Strong CYP3A Inhibitors

Current Selpercatinib Dosage	Recommended Selpercatinib Dosage	
	**Moderate CYP3A Inhibitor**	**Strong CYP3A Inhibitor**
120 mg orally twice daily	80 mg orally twice daily	40 mg orally twice daily
160 mg orally twice daily	120 mg orally twice daily	80 mg orally twice daily

Supplementary Table 5: Pralsetinib Dose Modifications when Combined with P-gp and Strong CYP3A Inhibitors

Current Pralsetinib Dosage	Recommended Pralsetinib Dosage
400 mg orally once daily	200 mg orally once daily
300 mg orally once daily	200 mg orally once daily
200 mg orally once daily	100 mg orally once daily

REFERENCES

1. Schlumberger, M., Elisei, R., Muller, S., Schoffski, P., Brose, M., Shah, M., Licitra, L., Krajewska, J., Kreissl, M.C., Niederle, B., et al. (2017). Overall survival analysis of EXAM, a phase III trial of cabozantinib in patients with radiographically progressive medullary thyroid carcinoma. Ann Oncol *28*, 2813-2819. 10.1093/annonc/mdx479.

2. Drilon, A., Rekhtman, N., Arcila, M., Wang, L., Ni, A., Albano, M., Van Voorthuysen, M., Somwar, R., Smith, R.S., Montecalvo, J., et al. (2016). Cabozantinib in patients with advanced RET-rearranged non-small-cell lung cancer: an open-label, single-centre, phase 2, single-arm trial. Lancet Oncol *17*, 1653-1660. 10.1016/S1470-2045(16)30562-9.

3. Wells, S.A., Jr., Robinson, B.G., Gagel, R.F., Dralle, H., Fagin, J.A., Santoro, M., Baudin, E., Elisei, R., Jarzab, B., Vasselli, J.R., et al. (2012). Vandetanib in patients with locally advanced or metastatic medullary thyroid cancer: a randomized, double-blind phase III trial. J Clin Oncol *30*, 134-141. 10.1200/JCO.2011.35.5040.

4. Wirth, L.J., Brose, M.S., Elisei, R., Capdevila, J., Hoff, A.O., Hu, M.I., Tahara, M., Robinson, B., Gao, M., Xia, M., et al. (2022). LIBRETTO-531: a phase III study of selpercatinib in multikinase inhibitor-naïve RET-mutant medullary thyroid cancer. Future Oncol. 10.2217/fon-2022-0657.

5. Drilon, A., Subbiah, V., Gautschi, O., Tomasini, P., de Braud, F., Solomon, B.J., Shao-Weng Tan, D., Alonso, G., Wolf, J., Park, K., et al. (2022). Selpercatinib in Patients With RET Fusion-Positive Non-Small-Cell Lung Cancer: Updated Safety and Efficacy From the Registrational LIBRETTO-001 Phase I/II Trial. J Clin Oncol, JCO2200393. 10.1200/JCO.22.00393.

6. Subbiah, V., Wolf, P.J., Konda, B., Kang, H., Spira, A., Weiss, J., Takeda, M., Ohe, Y., Khan, S., Ohashi, K., et al. (2022). Tumour-agnostic efficacy and safety of selpercatinib in patients with RET fusion-positive solid tumours other than lung or thyroid tumours (LIBRETTO-001): a phase 1/2, open-label, basket trial. Lancet Oncol. 10.1016/s1470-2045(22)00541-1.

7. Subbiah, V., Hu, M.I., Wirth, L.J., Schuler, M., Mansfield, A.S., Curigliano, G., Brose, M.S., Zhu, V.W., Leboulleux, S., Bowles, D.W., et al. (2021). Pralsetinib for patients with advanced or metastatic RET-altered thyroid cancer (ARROW): a multi-cohort, open-label, registrational, phase 1/2 study. Lancet Diabetes Endocrinol *9*, 491-501. 10.1016/S2213-8587(21)00120-0.

8. Griesinger, F., Curigliano, G., Thomas, M., Subbiah, V., Baik, C.S., Tan, D.S.W., Lee, D.H., Misch, D., Garralda, E., Kim, D.W., et al. (2022). Safety and efficacy of pralsetinib in RET fusion-positive non-small cell lung cancer including as first-line therapy: update from the ARROW trial. Ann Oncol. 10.1016/j.annonc.2022.08.002.

9. Subbiah, V., Cassier, P.A., Siena, S., Garralda, E., Paz-Ares, L., Garrido, P., Nadal, E., Vuky, J., Lopes, G., Kalemkerian, G.P., et al. (2022). Pan-cancer efficacy of pralsetinib in patients with RET fusion-positive solid tumors from the phase 1/2 ARROW trial. Nat Med *28*, 1640-1645. 10.1038/s41591-022-01931-y.
